# Homologous recombination deficiency in ovarian cancer: a review of its epidemiology and management

**DOI:** 10.6061/clinics/2018/e450s

**Published:** 2018-08-03

**Authors:** Renata Rodrigues da Cunha Colombo Bonadio, Rodrigo Nogueira Fogace, Vanessa Costa Miranda, Maria del Pilar Estevez Diz

**Affiliations:** Instituto do Cancer do Estado de Sao Paulo (ICESP), Hospital das Clinicas HCFMUSP, Faculdade de Medicina, Universidade de Sao Paulo, Sao Paulo, SP, BR

**Keywords:** Ovarian Cancer, Homologous Recombination Deficiency, PARP Inhibitors, BRCA Mutation

## Abstract

Ovarian cancer patients with homologous recombination deficiencies exhibit specific clinical behaviors, and improved responses to treatments, such as platinum-based chemotherapy and poly (ADP-ribose) polymerase (PARP) inhibitors, have been observed. Germline mutations in the BRCA 1/2 genes are the most well-known mechanisms of homologous recombination deficiency. However, other mechanisms, such as germline and somatic mutations in other homologous recombination genes and epigenetic modifications, have also been implicated in homologous recombination deficiency. The epidemiology and implications of these other mechanisms need to be better understood to improve the treatment strategies for these patients. Furthermore, an evaluation of various diagnostic tests to investigate homologous recombination deficiency is essential. Comprehension of the role of homologous recombination deficiency in ovarian cancer also allows the development of therapeutic combinations that can improve the efficacy of treatment. In this review, we discuss the epidemiology and management of homologous recombination deficiency in ovarian cancer patients.

## INTRODUCTION

In ovarian cancer, patients harboring BRCA 1/2 mutations exhibit different patterns of clinical behavior and respond to treatment differently. The BRCA gene plays a role in repairing DNA repair via homologous recombination (HR), and mutation of this gene leads to HR deficiency (HRD).

HRD can also occur due to other mechanisms, such as germline mutations, somatic mutations and epigenetic modifications of other genes involved in the HR pathway. Ovarian cancers with these alterations behave similarly to those with BRCA mutations, and this behavior is termed the “BRCAness” phenotype.

Using poly (ADP-ribose) polymerase (PARP) inhibitors in patients with HRD compromises two pathways of DNA repair, resulting in synthetic lethality. Recent studies have confirmed that the efficacy of PARP inhibitors is improved not only in ovarian cancers displaying germline or somatic BRCA mutations but also in cancers in which HRD is caused by other underlying etiologies.

In this review, we discuss how to evaluate HRD as well as the epidemiology and management of HRD in ovarian cancer.

### Homologous recombination deficiency and PARP inhibitors

DNA breaks are repaired via different mechanisms to protect the genome. For example, double-stranded breaks are repaired by HR and non-homologous end joining (NHEJ) 1. HR is more efficient at maintaining genomic stability because it uses a homologous template, whereas NHEJ is error-prone.

Unrepaired DNA damage can result in accumulated mutations and unregulated cell division, and HRD is thus related to cancer susceptibility 2,3. Moreover, large amounts of DNA damage can lead to cell apoptosis. However, when only HR is deficient, the activities of other DNA repair mechanisms can prohibit the accumulation of excessive DNA damage and apoptosis 2.

Base excision repair (BER) serves as another DNA repair mechanism that acts on single-stranded breaks, and members of the PARP protein family play essential roles in the BER mechanism. PARPs bind to single-stranded break sites and initiate the repair process, and these proteins are targeted in oncology via the use of PARP inhibitors.

As mentioned previously, HRD by itself does not always induce cellular apoptosis. However, when PARP inhibitors are used in HRD cells, impairment of these two DNA repair mechanisms together results in synthetic lethality. In other words, mutations occurring in one of two genes separately do not result in apoptosis, but the impairment of both genes simultaneously leads to cell death (synthetic lethality). In this situation, the accumulation of DNA damage might be sufficient to induce cell death (apoptosis), and clinical trials showing the benefits of PARP inhibitors in HRD cancers support this concept 4,5.

### Mechanisms implicated in homologous recombination deficiency

The most described etiology of HRD is the mutation of genes involved in HR repair. Mutations can occur in germline cells, which represent individual characteristics, or somatic cells, which is a trait of tumor cells.

Germline BRCA 1 and BRCA 2 mutations are the most well-known HRD etiology. Germline mutations are important not only for treatment decisions but also for the evaluation of cancer susceptibility and prevention strategies for the patients and their relatives. BRCA 1/2 are involved in hereditary breast and ovarian cancer syndrome, and numerous trials evaluating PARP inhibitors have been performed on patients presenting germline BRCA 1/2 mutations 5-8. Patients without BRCA 1/2 mutations presented similar clinical behaviors and responses to PARP inhibitors 4,9, and these patients define the “BRCAness” phenotype 10,11. The mechanisms underlying BRCAness are varied and include somatic BRCA 1/2 mutations and germline or somatic mutations in other genes related to HR repair.

Another possible etiology of HRD is the epigenetic modification of HR genes, such as methylation of the BRCA 1 promoter. Gene expression signatures present in germline BRCA1 mutations were also observed in BRCA1-methylated cancers 12. However, the implication of epigenetic modifications in HRD remain controversial. While Cunningham et al. reported a survival advantage in patients with BRCA 1 promoter hypermethylation compared with BRCA wild-type patients 13, other researchers found no survival advantage 14,15 or worse survival for patients with the methylated phenotype 16.

### How to evaluate homologous recombination deficiency

HRD can be tested using three main strategies:

- Germline mutation screening of genes related to HR repair;
- somatic mutation screening of genes related to HR repair; and
- evaluation of a genomic scar, which represents the genomic instability secondary to HRD. An HRD score can be calculated based on the loss of heterozygosity (LOH), telomeric allelic imbalance, and large-scale transitions.

Germline mutation screening can be performed using next generation sequencing (NGS) analysis of DNA from blood, which has the advantage of being easy to obtain. Moreover, the identification of a germline mutation allows the possibility of genetic counseling.

Somatic mutation screening, on the other hand, is performed on DNA from tumor samples. This analysis can evaluate any mutation (germline and/or somatic) in HR genes and is thus a broader evaluation, which is helpful for defining treatment strategies, such as the use of PARP inhibitors. However, when a mutation is identified with this strategy, germline analysis of normal cells is still necessary to determine whether the mutation is germline or somatic (present in only the tumor) 17. Limitations of somatic screening include the variability of tumor samples available and intratumoral heterogeneity, which potentially compromises the representativeness of the sample.

Finally, HRD can be assessed in a more functional way. When HRD is present, genomic alterations accumulate, and allelic imbalances can result in a “genomic scar”, allowing the investigation of HRD regardless of the underlying genetic or epigenetic mechanism responsible. A high LOH (≥14-16%), for example, suggests the presence HRD. In the ARIEL2 trial, which evaluated rucaparib in platinum-sensitive recurrent ovarian cancer, LOH-high was defined by NGS using a cutoff of 14%. The results showed that patients with BRCA 1/2 wild-type and LOH-high benefited from rucaparib 9. LOH can also be evaluated together with telomeric allelic imbalance and large-scale transitions to generate an HRD score (MyChoice^®^ HRD test, Myriad Genetics Inc., Salt Lake City, Utah). Patients determined to have HRD (defined as any tumor that scored ≥42 on the MyChoice^®^ HRD test) benefited from maintenance niraparib in the NOVA trial 4.

Despite the ability of NGS to assess many genes during germline or somatic mutation screening, the implications of some mutations remain unknown. Moreover, NGS cannot evaluate HRD due to other etiologies, such as epigenetic modifications. Thus, functional evaluations of HRD can help overcome these limitations.

In conclusion, each of these tests have different properties and can be used in a complementary manner.

### Epidemiology of HRD in ovarian cancer

Approximately 41-50% of ovarian carcinomas are estimated to exhibit HRD 17,18. However, the frequency of HRD varies according to the method utilized for its evaluation (germline mutations, somatic mutations or HRD score) and histological subtype. Table 1 shows the frequencies of HRD in different studies according to the histological subtype.

Pennington et al. 19 found HR gene germline mutations in 24% of patients with epithelial ovarian cancer and somatic mutations in 9% of these patients. Elvin et al. 18 evaluated the presence of BRCA mutations or LOH in different histological subtypes. The serous subtype was associated with a higher prevalence of HRD, with 43.8% of the patients presenting BRCA mutations (BRCAmut, 18.7%) or BRCA wild-type/LOH-high (BRCAwt/LOH-high, 25.1%). Other epithelial ovarian carcinomas also exhibited elevated proportions of HRD which occurred in 37.6% of endometrioid (12.6% BRCAmut and 25% BRCAwt/LOH-H), 23.5% of carcinosarcoma (8.2% BRCAmut and 15.3% BRCAwt/LOHH) and 12.6% of clear cell histologies (4.7% BRCAmut and 8.9% BRCAwt/LOHH). The mucinous subtype, however, exhibited no BRCA mutations, and only 8.1% of the patients presented with BRCAwt/LOH-H. Upon specifically evaluating the presence of somatic mutations, Aghajanian et al. found similar prevalences of somatic mutations in high-grade serous ovarian carcinoma (HGSOC) and other histologies (16% *vs* 18%, respectively, *p*=0.07). Once again, no somatic mutations in HR genes were observed in mucinous ovarian cancer.

In relation to high-grade versus low-grade serous carcinoma, Norquist et al. 21 found a significant difference in the germline and somatic mutation rates of HR genes, which were 10.9% for low-grade *versus* 27% for HGSOC (odds ratio (OR), 0.33; 95% confidence interval (CI), 0.1-0.8; *p*=0.02).

Regarding the specific genes compromised, in an evaluation of HR gene mutations in ovarian cancer patients who participated in the GOG 218 and GOG 262 trials, Norquist et al. 21 showed germline or somatic mutations in BRCA1 gene in 12.3% of the cases, in BRCA2 gene in 6.5% and in other non-BRCA HR genes in 6.8%. Elvin et al. 18 reported similar results, with mutations in BRCA 1 gene in 11.6% of the cases and BRCA 2 gene in 5.7%.

BRCA mutations occur more frequently in HGSOC, with 20% of these patients presenting germline or somatic mutations in BRCA 1 or BRCA 2 21. However, in the Norquist et al. study 21, other histologies also presented considerable rates of BRCA 1 or 2 mutations (approximately 9% for endometrioid ovarian cancer, 11% for clear cell ovarian cancer and 8% for low-grade serous ovarian cancer). Alsop et al. 22 exclusively evaluated the frequencies of germline BRCA 1 and BRCA 2 mutations, finding mutations in 17% of patients with HGSOC, 8.4% of patients with the endometrioid histology and 6.3% of patients with the clear cell histology. Somatic BRCA 1 and BRCA 2 mutations occur less often, with prevalences of 2-5% and 2-3%, respectively 14,19.

The frequency of changes (mutation, deletion or amplification) in each non-BRCA HR gene is much lower and more heterogeneous. Table 2 lists the genes implicated in HR repair and the frequencies of germline mutations reported in different studies. Table 3 describes the frequencies of somatic gene changes. NGS was utilized in all the studies described in the tables. Blood samples were utilized in trials that evaluated germline mutations, and tumor samples were utilized in trials evaluating somatic gene mutations.

### Treatment of ovarian cancer with HRD

HRD carcinomas exhibit an increased responsiveness to cytotoxic chemotherapy, especially platinum agents, in different treatment lines 19,. Platinum agents act via directly damaging DNA, and when HRD is present, the reduction of DNA repair increases the accumulation of DNA damage, leading to apoptosis. Pennington et al. showed that somatic BRCA 1/2 mutations and mutations in other HR genes predict platinum responsiveness and positively impact overall survival, similar to germline BRCA 1/2 mutations 19.

Regarding PARP inhibitors, their benefit in HRD was first shown in patients with BRCA 1/2 mutations. In a phase I trial, the activity of olaparib was evaluated in heavily pretreated patients (mainly ovarian and breast cancer patients) 26. Twelve of the 23 patients harboring BRCA mutations presented a response or stable disease for at least 4 months, while no response was observed in patients without BRCA mutations.

A benefit of olaparib in patients with BRCA mutations was also suggested in the phase II study 19 trial 27. This trial evaluated olaparib maintenance in platinum-sensitive patients with or without BRCA mutations and showed improved progression free survival (PFS) in comparison to that of patients receiving the placebo (8.4 months versus 4.8 months; hazard ratio, 0.35; 95% CI, 0.25-0.49; *p*<0.001). However, the benefit was greater in patients with BRCA mutations (11.2 vs 4.3 months; hazard ratio, 0.18; 95% CI, 0.10-0.31; *p*<0.0001) than in BRCA wild-type patients (7.4 *vs* 5.5 months; hazard ratio, 0.54; 95% CI, 0.34-0.85, *p*=0.0075). Furthermore, in a post hoc analysis, when excluding patients who crossed over to olaparib after progression, an improved overall survival with olaparib was observed in the group with BRCA mutations (hazard ratio, 0.52; 95% CI, 0.28-0.97) 28.

Another single-arm phase II study evaluated olaparib in patients with germline BRCA mutations previously treated with at least three lines of chemotherapy 5. The results were impressive in this heavily pretreated population, with a response rate of 31.1% in ovarian cancer patients, a median PFS of 7 months and a median overall survival of 16.6 months. These results lead to the Food and Drug Administration (FDA) approval of olaparib for this scenario.

Recently, results of the phase III SOLO 2 trial were published, showing that patients with BRCA mutations that had previously received at least two lines of chemotherapy benefited from olaparib maintenance after response to platinum-based chemotherapy for the treatment of relapsed ovarian cancer. The risk of progression was reduced by 70%, with an absolute gain in PFS of 13.6 months (median PFS of 19.1 months with olaparib *versus* 5.5 months with the placebo; hazard ratio, 0.3; 95% CI, 0.22-0.41; *p*<0.0001).

Another PARP inhibitor, niraparib, also demonstrated efficacy in patients with ovarian cancer with or without BRCA mutations. The NOVA trial showed that as a maintenance therapeutic, niraparib improves the PFS of platinum-sensitive patients 4. In that trial, the presence of BRCA mutations and HRD determined using the Myriad Genetics HRD score were investigated. While a benefit was observed in all subgroups, the PFS of patients with BRCA mutations (21.0 months *vs* 5.5 months; hazard ratio, 0.27; 95% CI, 0.17-0.41) and BRCA wild-type/HRD-high (20.9 months *vs* 11.0 months; hazard ratio, 0.27; 95% CI, 0.08-0.90) was increased to a greater extent than that of BRCA wild-type/HRD-low patients (6.9 months *vs* 3.8 months; hazard ratio, 0.58; 95% CI, 0.36-0.92).

The phase II Ariel 2 trial also confirmed the benefit of PARP inhibitors to patients with HRD in general 9. In this trial, rucaparib was used in advanced ovarian cancer patients previously treated with two or more lines of chemotherapy (regardless of their platinum sensitivity). HRD was assessed by evaluating both BRCA germline mutations and LOH. Once again, the response rate (RR) and PFS were higher in BRCA germline mutation carrier patients (RR, 69%; PFS, 12.8 months; hazard ratio, 0.27; 95% CI, 0.16-0.44; *p*<0.0001) and BRCA wild-type LOH-high patients (RR, 39%; PFS, 5.7 months; hazard ratio, 0.62; 95% CI, 0.42-0.9; *p*=0.011) than in BRCA wild-type/LOH-low patients (RR, 11%; PFS, 5.1 months).

The phase III Ariel 3 trial showed that rucaparib also improved the PFS as a maintenance therapeutic in ovarian cancer patients in comparison with the placebo after treatment with at least two lines of platinum-based therapy with response to the last treatment 29. PFS was improved in the three nested cohorts: patients with BRCA mutations (median PFS, 16.6 months *vs* 5.4 months; hazard ratio, 0.23; 95% CI, 0.16-0.34; *p*<0.0001), patients with HRD (including BRCAmut and BRCAwt/high-LOH carcinomas) (median PFS, 13.6 months *vs* 5.4 months; hazard ratio, 0.32; 95% CI, 0.24-0.42; *p*<0.0001) and the intention-to-treat population (median PFS, 10.8 months *versus* 5.4 months; hazard ratio, 0.36; 95% CI, 0.30-0.45; *p*<0.0001). In a non-nested subgroup analysis, the absolute gain in median PFS was 4.3 months for BRCA wild-type patients with LOH-high (9.7 months *vs* 4 months; hazard ratio, 0.44; *p*<0.001) and 1.3 months for those with LOH-low (6.7 months *vs* 5.4 months; hazard ratio, 0.58; *p*=0.0049).

In conclusion, the trials show that the benefits of PARP inhibitors extend beyond BRCA 1/2 mutations. Patients with HRDs of different etiologies might benefit from these drugs, increasing the number of patients who might benefit from these treatments. Some studies also showed a statistically significant benefit for the PFS of patients with HR proficiency, but the clinical relevance of the gain in this scenario was smaller.

### Perspectives

Studies are ongoing to investigate whether combinations of PARP inhibitors and other drugs might improve their efficacy in patients with or without HRD.

As mentioned previously, when HRD is present, the use of PARP inhibitors induces synthetic lethality. In patients without HRD, using drugs in combination might exert a similar effect, defined as ‘contextual' synthetic lethality 30.

Hypoxic conditions, for example, appear to downregulate DNA repair and generate genomic instability 30,31. Thus, the combination of antiangiogenic agents and PARP inhibitors represents a potential mechanism underlying contextual synthetic lethality. In a phase II trial, olaparib was combined with the VEGFR inhibitor cediranib, and an improved in PFS was observed (17.7 months *versus* 9 months with olaparib alone; hazard ratio, 0.42; 95% CI, 0.23-0.76; *p*=0.005) 32. Upon subgroup analysis, patients with HR proficiency benefited the most from the synergism of the two drugs (PFS of 16.5 *vs* 5.7 months with olaparib alone; hazard ratio, 0.32; *p*=0.008). Patients with germline BRCA mutations had a good response to olaparib alone, as expected, and an improvement trend in PFS was observed with the combination (PFS of 19.5 months *vs* 16.5 months with olaparib alone).

For patients with HRD who develop resistance to PARP inhibitors, the association of VEGFR and PARP inhibitors represents a potential strategy to overcome resistance. Thus, a study evaluating the combination of cediranib and olaparib in advanced ovarian cancer after progression on a PARP inhibitor is currently ongoing (ClinicalTrials.gov, NCT02681237).

PI3K inhibitors are also associated with decreased HR repair. Preclinical studies showed that PI3K inhibitors decrease the expression of RAD51 and are synergistic with olaparib 33,34.

In addition to PI3K and VEGFR inhibitors, other agents that decrease DNA repair with the potential to function synergistically with PARP inhibitors include inhibitors of CHK1, ATR, Wee, BET 35-39. Preclinical studies have shown promising results when these agents are used in combination with PARP inhibitors.

Furthermore, cytotoxic chemotherapy might potentiate the effect of PARP inhibitors via the association of DNA damage and inhibition of DNA repair. In a phase I trial, the combination of olaparib and carboplatin yielded an overall RR of 44% in patients with germline BRCA mutations and ovarian cancer 40.

Another important point to consider is that adaptive resistance develops over time when a drug is used as monotherapy, and combination therapy could help avoid or retard the development of adaptive resistance.

Different mechanisms are implicated in the resistance to PARP inhibitors. In BRCA-mutated tumors, the development of secondary reversion mutations that restore BRCA function and HR activity appears to be an important mechanism underlying resistance 41,42.

Resistance might also occur upon the activation of signaling cascades implicated in tumorigenesis, such as the PI3K/AKT and RAS/MAPK pathways 34,43.

As mentioned previously, PI3K inhibitors improve the activity of olaparib 33,34, and PARP and MEK inhibitors also function synergistically when used in combination both *in vitro* and *in vivo* 43. RAS mutant lines, for example, are resistant to PARP inhibitors but sensitive to the combination of PARP and MEK inhibitors 43. A study on using the MEK inhibitor selumetinib and olaparib in combination to treat RAS-activated tumors is currently ongoing (ClinicalTrials.gov, NCT03162627).

Importantly, the combinations described above are potential treatment strategies for improving the efficacy of PARP inhibitors in patients with HR proficiency and patients with HRD that acquire resistance to PARP inhibitors.

The increase in drug efflux by P-glycoproteins (P-gp) also leads to PARP resistance, which was reversed by the coadministration of the P-gp inhibitor tariquidar in a preclinical study 44. Moreover, differences exist between PARP inhibitors, and while olaparib appears to be a substrate of P-gp, veliparib does not 45.

Alterations in PARP expression might also play a role in PARP resistance, and these effects may vary among different PARP inhibitors. While olaparib and veliparib specifically inhibit PARP1 and PARP2, niraparib, rucaparib and talazoparib inhibit a broader range of PARP enzymes 46. Thus, the possibility exist that after progression on one PARP inhibitor, another could still be active. However, this hypothesis needs to be investigated further.

Finally, the combination of PARP inhibitors with immunotherapy is also being studied 47. BRCA 1/2-mutated HGSOC exhibits a higher mutational load, more tumor-specific antigens, more tumor-infiltrating lymphocytes and higher PD-1 and PD-L1 expression in tumor-associated immune cells than HR-proficient HGOSC 48. These findings suggest that BRCA 1/2-mutated HGSOC may be more sensitive to PD1/PL-L1 inhibitors. Furthermore, the inhibition of DNA repair pathways propagating DNA damage and neoantigen formation could improve the activity of immunotherapy.

## CONCLUSIONS

The prevalence of HRD is high in ovarian cancer. Further understanding HRD and recognizing the existence of the BRCAness phenotype could lead to a broader group of patients benefiting from PARP inhibitors.

The best strategy to evaluate HRD still needs to be defined. Germline or somatic mutations can be assessed using NGS, while genomic instability can be determined by evaluating the LOH, telomeric allelic imbalance, and large-scale transitions. Each of these options has advantages and disadvantages, and they should be used in a complementary manner. Future studies on PARP inhibitors should continue to validate the clinical utility of these strategies to assess HRD.

Finally, the combination of PARP inhibitors with other drugs is promising. Currently, contextual synthetic lethality and strategies to overcome adaptive resistance have been studied using a combination of PARP inhibitors and other drugs, such as cytotoxic chemotherapy, angiogenesis inhibitors, MEK inhibitors and immunotherapy. These combinations might improve the efficacy of PARP inhibitors even in patients without HRD, extending the benefit of these drugs even further. We eagerly await further results from these studies.

## AUTHOR CONTRIBUTIONS

Bonadio RR, Fogace RN, Miranda VC and Dis MP contributed to the conception and design of the study, data collection, data analysis, and manuscript writing and revision.

## Figures and Tables

**Table 1:** Frequency of homologous recombination deficiency according to the histological subtype.

Method	Elvin et al. (N=4114) 18	Norquist et al. (N=1915) 21	Pennington et al. (N=367) 19
	BRCA + LOH-H	HR gene mutations	HR gene mutations
**Serous**	43.8%	27%	31%
**Endometrioid**	37.6%	23.8%	27%
**Carcinosarcoma**	23.5%	-	33%
**Clear Cell**	13.6%	21.4%	26%
**Epithelial NOS**	47.7%	-	-
**Mucinous**	8.1%	28.6%	0%

LOH-H: Loss of heterozygosity; HR: homologous recombination; NOS: not otherwise specified.

**Table 2:** Frequency of germline mutations in ovarian carcinoma.

(N)	TCGA 14 (316)	Pennington et al. 19 (390)	Cunningham et al. 13 (899)	Harter et al. 49 (522)	Norquist et al. 50 (1915)	Yates et al. 51 (299)
**BRCA1**	8.5%	13.4%	3.5%	15.3%	9.5%	9%
**BRCA2**	6.3%	4.6%	3%	5.6%	5.1%	5.4%
**EMSY**						
**PTEN**				0%		
**RAD51C**		0.7%	3%	2.5%	0.6%	1%
**RAD51D**		1%		0.6%	0.6%	
**RAD50**				0.2%	0.2%	
**ATM/ATR**				0.4%	0.6%	0.5%
**FANC**				0.7%		
**BARD1**		0.5%		0%	0.2%	0.5%
**BRIP1**		1%		0.4%	1.4%	2.5%
**CHEK1**		0.25%		0.2%		
**CHEK2**		0.7%		0.6%	0.6%	
**FAM175A**		0.5%		0.2%	0.2%	
**NBN**		0.25%		0.4%	0.5%	0.5%
**PALB2**		0.5%		1.1%	0.6%	0.5%
**MRE11A**				0.4%	0.1%	
**MMR**				0.6%	0.5%	
**TP53**				0%	0.3%	

FANC: Fanconi anemia complementation group; MMR: mismatch repair genes.

**Table 3:** Frequency of somatic gene changes (mutation, deletion or amplification) in ovarian carcinoma.

(N)	TCGA 14 (316)	Pennington et al. 19 (390)	Cunningham et al. 13 (279)	Hahnen et al. 52 (431)	Aghajanian et al. 20 (260)
**BRCA1**	3.2%	4.9%	2%	3%	4.4%
**BRCA2**	2.9%	1.5%	1.4%	1.4%	2.2%
**EMSY**	8%				
**PTEN**	7%				4.4%
**RAD51C**	0.3%	0.3%			
**RAD51D**				0.2%	
**RAD50**	0.6%				
**ATM/ATR**	2%	0.8%		0.2%	2.2%
**FANC**	5%			0.2%	0.3%
**BARD1**					0.6%
**BRIP1**		0.5%			0.6%
**CHEK1**	0%				0.3%
**CHEK2**	0.3%	0.8%			0.3%
**FAM175A**					
**NBN**					0.3%
**PALB2**				0.2%	0.3%
**MRE11A**		0.3%			
**MMR**	0.4%				
**TP53**					

FANC: Fanconi anemia complementation group; MMR: mismatch repair genes.
